# Adherence to recommended hospital waste management practices by healthcare workers at Murtala Muhammad Specialist Hospital Kano, Nigeria

**DOI:** 10.11604/pamj.2023.44.124.34618

**Published:** 2023-03-14

**Authors:** Zainab Muhammad Yakubu, Isah Mohammed Bello, Fatimah Tsiga-Ahmed

**Affiliations:** 1 Department of Community Medicine, Bayero University, Kano State, Nigeria,; 2 World Health Organization (WHO), Inter-Country Support Team Office for East and Southern Africa, P.O. Box 5160, Harare, Zimbabwe

**Keywords:** Medical waste management, health care workers, waste management practice, Murtala Muhammad Specialist Hospital, Kano, Nigeria

## Abstract

**Introduction:**

the aim of this study was to determine what proportion of patients with confirmed esophageal cancer at the largest hospital in the country were recorded in the Zambia National Cancer Registry (ZNCR).

**Methods:**

we reviewed esophageal cancer records at the University Teaching Hospital (UTH) and ZNCR, between 2015 and 2017. Using Stata version 15, data were summarised and the Kruskal-Wallis was used to compute comparisons, Kaplan-Meier curves for survival estimates and Cox regression for associated factors.

**Results:**

included in the final analysis were records for 222 patients with confirmed esophageal cancer and of these 51/222 (41%) were appearing in the ZNCR. The mean age of the patients was 56.2 years (SD, 13.0) and only 2/222 (1%) were confirmed alive at the time of data analysis. The median time from endoscopic diagnosis to histological confirmation was 12.5 days (IQR 7.5 - 21.5) and arrival at the Cancer Diseases Hospital (CDH) for treatment was 20 days (IQR 10 - 34). The overall median survival time in the study was 259 days (CI 95%; 151 - 501). Age, sex, time to diagnosis, histological classification and grade of tumour did not show any evidence of predicting survival in both the univariate and multivariable cox regression model (p>0.05).

**Conclusion:**

a significant proportion of esophageal cancer cases seen at UTH were not included in the national registry suggesting that official figures for the prevalence of esophageal cancer in Zambia are underestimated. There is an urgent need to improve the collection of data on esophageal cancer in Zambia.

## Introduction

Hospital waste management is a global problem of immediate concern, due to rapid increase in the hospital acquired infections both by the public and the health personnel. Most of the hospital generated waste is potentially infectious and therefore, spreads infection amongst the community causing a major health problem. It had been noted that in general, about 85% of waste materials from healthcare facilities belong to the general waste category, while the remaining 15% would consist of highly infectious or toxic radioactive materials [1]. Therefore, the hospital waste management assumes utmost importance in the present public health scenario, where the emergence and re-emergence of the infectious diseases are a major public threat. The wastes generated by the hospitals add to the community waste, thereby putting the load on the already scarce resources [2]. Although waste is generated from anywhere such as the home, office, industry, agriculture, school, living things and healthcare establishments, but wastes from healthcare poses greater threat due to its hazardous nature and disease transmission characteristics [3]. Hazardous waste exposure can occur as a consequence of an accident, ignorance, nonchalance, or willful carelessness on the side of waste handlers, from water, food, home items, breast milk, and foetus in the womb. The many categories of hazardous waste each have their own detrimental health effects, which may differ or be comparable. Some wastes' detrimental consequences may not be visible while they are being utilized and/or before they are thrown. People may be exposed during the manufacture, shipping, distribution, and/or use of a product, for example. Most chemicals and cytotoxic medications are prime examples of items that are hazardous throughout their entire life cycle and disposal [3].

Hospital waste has a significant effect on the environment, wastes in the form of sharp objects, discarded human tissues during surgical operations, blood tissues and patients´ vomitus, chemical and pharmaceutical materials, which depending on the level of their associated hazard, are meant to be disposed according to some approved international standards are disposed along with domestic wastes into landfills or municipal´s open waste dumpsites. This increases the risk of human contacts with these hazardous and highly infectious waste products and exposes the entire population to several forms of environmental pollution because some waste collectors could have access to these landfill sites and open waste dumpsites. The likelihood of human contact with highly infectious hospital waste increases, when domestic wastes are disposed along with hospital waste [1]. Refuse is thrown onto roadways, spread on pedestrian walkways, or even dumped into gutters, and during rainy season, the situation is exacerbated as the gutters get blocked, thereby becoming stagnant, producing ideal conditions for mosquitoes and vector-borne illnesses such as malaria and cholera [4].

Many hospitals, clinics, and health centers' inadequate segregation, handling, and disposal policies are likely reflective of practices throughout Nigeria, posing major health risks to those living near healthcare institutions [3]. However, the nature and quantity of healthcare waste generated, as well as the institutional practices in regard to sustainable methods of hospital waste management are poorly examined and documented in our healthcare institutions [5]. Contamination of water supply from untreated hospital waste can also have devastating effects. If infectious stools or bodily fluids are not treated before disposal, they can cause and spread epidemics. For example, because sewage treatment in Africa is almost non-existent, the absence of proper sterilization procedures is thought to have increased the severity and size of cholera epidemics in Africa over the last decade [6]. The sustainable management of Hospital Waste has continued to generate increasing public interest due to the health problems associated with exposure of human beings to potentially hazardous wastes arising from hospital waste. Presently, considerable gap exist with regard to the assessment of hospital waste management practices, particularly in Nigeria and in several other countries in sub - Saharan Africa [5]. It is widely accepted that the management of solid waste is a global problem, which is even more pronounced in developing countries such as Nigeria, where solid waste management is a major concern [7].

The importance of proper hospital waste management is universally acknowledged by international organizations e.g. the World Health Organization (WHO) which recommends 80% of waste generated should be non-infectious and can be recommended to join the municipal waste stream, while 20% is the infectious waste that requires unique waste treatment methods [8], as ideal Hospital Waste Disposal practices to prevent and control its negative effect on health and environment. Also, assessment of hospital waste disposal practices as recommended by WHO is rarely put into research in Nigeria, especially in the Northern part. However, the nature and quantity of healthcare waste generated as well as the institutional practices in regard to sustainable methods of hospital waste management including waste segregation and waste recycling are poorly examined and documented in our healthcare institutions despite the health risks posed by improper handling of healthcare wastes [6]. The developed countries have properly organized infrastructure of hospital waste disposal. A fully trained staff oversees the management of different waste disposals such as segregation, internal transit, and final disposal. Whereas in developing countries, the situation is not the same, there is a lack of awareness regarding segregation, collection, storage and, transportation and waste, as most health workers have little awareness of the hazards associated, have poor disposal techniques, and also lack awareness of health policy and laws [8]. The average generation rate of medical waste in investigated hospitals in Lagos, Nigeria ranged from 0.562 kg/bed/day to 0.670 kg/bed/day. Infectious waste accounted for 26% to 37% of this volume [9].

In Nigeria, practice, and adherence to the recommended best standards are poorly supervised by assigned bodies at both local and the national level [7]. Likewise, the international policy responsible for the proper management, treatment, and disposal of waste has remained on paper, and is yet to be implemented in many States. This study is aimed to find gaps in the current practices of healthcare waste disposal in Northern Nigeria compared with International best practices and recommend ways of bridging these gaps considering the current economic and technological realities in the country, using Murtala Muhammad Specialist Hospital in Kano as a case study. It will determine the level of adherence to hospital waste management practices by healthcare workers at Murtala Muhammad Specialist Hospital Kano and identify factors that would influence adherence amongst healthcare workers in the hospital.

## Methods

**Study area:**Kano is the capital of Kano State located in the North-western part of Nigeria. The State is bounded to the North by Jigawa and the Katsina States, to the South by Kaduna and the Bauchi States, to the West by Kaduna and the Katsina States and finally East of the State by Jigawa and Bauchi States. The State was found to have a population of 9,410,288 based on the 2006 National Population and Housing Census, which makes it one of the most populous States in the country, and using a growth rate of 3.1% per annum, Kano State, had a projected total population of 11,215,688 in 2012 and 13,065,294 in 2017 [10]. The population of the State is predominantly. Kano State is made up of 44 local government areas (LGAs).

**Study site:**Murtala Muhammad Specialist Hospital is located within the ancient city walls of Kano. The Hospital was established in 1927, initially called City Hospital, with a capacity of 16 beds at the time. It was renamed after the former Nigerian Head of State, General Murtala Ramat Muhammad in 1976. It became a Specialist Hospital in 1987 and is located eastward 400 m away from Kofar Mata and westward about 700 m from the emir´s palace. As of March 2018, the hospital has a staff strength of 1,656, this number includes all health workers and non-health workers. The Hospital currently has an official bed capacity of 826, Twenty (20) departments, the largest being the department of medicine, 30 wards and units, 9 operating theatres, 14 clinics, it is also an NHIS accredited hospital. Patients are seen all through the week, especially in patients, but outpatients are seen mainly from Monday to Friday. There is high patient turnover, far exceeding the required healthcare providers to patient ratio. This is because MMSH serves as a referral centre not only for the State but also for some parts of Northern Nigeria and some neighbouring countries like the Niger Republic and Cotonou [11].

**Study design/population:**the design of the study is descriptive cross-sectional that utilized mixed concurrent methods of data collection, consisting of survey questionnaires (quantitative) and in-depth interviews (qualitative). These methods were used to complement each other; the qualitative data was used to cross-validate the quantitative results. A simple random sampling technique for quantitative data involving two stages was used for the selection of respondents, while purposive sampling was used for the selection of key informant interview (KII) participants. The Study population is health professionals (Doctors, Nurses, Medical Lab scientists, CHEWs), hospital cleaners, and heads of the environmental health department.

**Sample size estimation:**the minimum sample size for quantitative data was estimated using Fisher´s formula for cross-sectional study below [12].


$$n=\frac{Z^{2}pq}{d^{2}}$$

Where: n = minimum sample size required, Z = value of the Standard normal deviate at 95% level of significance = 1.96. p = expected prevalence or proportion from previous study. Using prevalence of 35%, a study of hospital waste generation and management practice in south east Nigeria [13]. q = 1 - p = complementary probability. P = 0.35, then, q = 1 - 0.35 = 0.65, Z = 1.96, d = 0.05, d = degree of precision (d = 0.05). n = (1.96)^2^x 0.35 x 0.65 = 350(0.05)^2^. Using adherence rate of 35% from previous study conducted in South-East Nigeria [13]. n = 350. To account for non-response, the sample size was increased by 10%. = 10% of 350, Hence 0.1 x 350 = 35. Therefore, final sample size n = 350 + 35 = 385.

**Data collection method:**an interviewer-administered questionnaire was used for this study to collect quantitative information by trained research assistants. The questionnaires were distributed to the selected departments based on the allocated number of respondents. The filled questionnaires were checked during data collation and collection, with data editing and checking to exclude incomplete, inaccurate, and inconsistent data before analysis. Data was entered manually into an Excel sheet, and analysed using IBM SPSS version 20.0, statistically significant associations were set at (p<0.05). The results were presented in tables and charts. The information from tape recordings of qualitative data was transcribed verbatim including pauses and interruptions, merged with the field notes and analysed manually. Transcribed data were then reviewed, with each interview comment labelled. All connected themes in the data were thoroughly reviewed, organized, and interpreted, and further triangulated with results of quantitative data using thematic analysis.

**Ethical considerations:**ethical approval was sought for this study from Health Research Ethics Committees of Kano State Ministry of Health (MOH/Off/797/T.I/1296). Informed consent was obtained from the respondents after explaining the study goals, with guarantee of confidentiality of their answers.

**Limitations of the study:**adherence was assessed via self-reporting, and no confirmatory checklist or independent observation was used to confirm the actual adherence to waste management. Nonetheless, a minimal effect of this is expected as participants were assured of confidentiality.

## Results

Three hundred and ninety (390) questionnaires were administered, and 371 questionnaires were completed and returned, giving a response rate of 95.13%. Some of the questionnaires were not returned due to incomplete information, unavailability of staff, and misplacement of the questionnaires by some respondents. Most of the respondents were females (54%), and half of the respondents 197 (53%) were between 18 to 28 years, over two-thirds (70%) of the respondents were married, and the majority 260 (70%), had been in service for less than 5 years. A significant number of the respondents, 255 (69%) were Hausa/Fulani tribe, and 344 (93%) of the respondents were Muslims (Table 1). Table 2 shows that all the components of knowledge are statistically significant (p<0.05), except for the disposal of objects that can cause punctures or cuts (p=0.28). The knowledge of hospital waste generation has been categorized into good and poor, with the majority of the respondents having a good general knowledge of what hospital waste is (89%), knowledge of how the waste should be disposed of showed that 62% of the respondents have good knowledge with 38% having poor knowledge. The correct sequence of waste management, disposal of objects exposed to blood capable of causing punctures or cuts, and disposal of infectious non-biodegradable materials showed fair knowledge with 40%, 47%, and 47% respectively, while disposal of infectious biodegradable materials, colour code for normal and autoclaved waste showed poor knowledge with 38%, 25%, and 20% respectively.

**Table 1:** summary of socio-demographic characteristics of respondents

Variable	Frequency (n= 371)	Percentage
**Age group (years)**		
18 - 28	156	42
29 - 38	197	53
39 - 48	18	5
Mean/STD age	28.8/5.12	
Cadre		
Doctor	24	6
Nurse	148	40
Others*	199	54
Tribe		
Hausa/fulani	255	69
Non hausa/fulani	116	31
**Marital status**		
Single	95	26
Married	258	70
Divorced	14	4
Widowed	4	1
Religion		
Islam	344	93
**Christianity**	27	7
Gender		
Male	170	46
Female	201	54
**Years in service**		
1 – 5 years	260	70
5 – 10 years	95	26
> 10 years	16	4

Others*: community health workers, lab attendants, environmental health officers and technicians etc

**Table 2:** knowledge of hospital waste generation categorization

Knowledge of waste management	Good knowledge (n=371)	Poor knowledge (n=371)	Test statistic (x^2^)	p-value
Correct sequence of hospital waste management	331 (89)	40 (11)	228.2	0.01*
How hospital waste should be disposed	230 (62)	141 (38)	21.3	0.01*
Recommended practices for hospital waste disposal	148 (40)	223 (60)	28.2	0.01*
How objects capable of causing punctures or cuts, that may have been exposed to blood or body fluids should be disposed of	175 (47)	196 (53)	1.18	0.28
The colour code for the hospital waste to be autoclaved/disinfected	92 (25)	279 (75)	94.25	0.01*
The colour code for disposal of normal waste from the hospital waste	76 (20)	295 (80)	129.2	0.01*
How infectious non-biodegradable (gloves, IV set, syringes, nylon sutures etc) are disposed of	164 (44)	207 (56)	4.98	0.03*
How infectious biodegradable (extracted teeth, human tissues, membranes, cotton dressing, suture materials like black braided silk, vicryl etc) are disposed of	140 (38)	207 (56)	12.93	0.03*

* Statistically significant difference

Knowledge of waste management has been categorized based on the cadre of the respondents (doctors, nurses, and other health workers), which showed that 79%, 74%, and 84% of doctors, nurses, and other HCW respectively, have understood what waste management all is about, and 38%, 34%, and 30% know how it should be disposed of correctly. The result also showed that knowledge of the correct sequence of waste management in the doctor´s category is 63%, nurses have a 30%, and other HCW with 54%. Knowledge on how different objects should be disposed of according to the standard practices shows that doctors have 83%, nurses with 55%, and other HCW with 48%. The knowledge of different color-coding according to the method of disposal also reveals that the doctor´s category has 54%, nurses 19%, and other HCW 23%. Furthermore, 54%, 39%, and 48% of doctors, nurses and other HCW knows how to dispose of infectious and non-degradable objects, while 54%, 40%, and 34% of the doctors, nurses, and other HCW knows about infectious biodegradable materials emanating from the hospital waste (Table 3).

**Table 3:** knowledge of hospital waste generation based on cadre of respondents

Knowledge of waste management	Doctors (n=24)	Nurses (n=148)	Other HCW (n=199)
Meaning of hospital waste	19 (79)	110 (74)	167 (84)
How hospital waste should be disposed of	9 (38)	51 (34)	60 (30)
Correct sequence of hospital waste management	15 (63)	44 (30)	67 (44)
Disposal of objects that can cause punctures or cuts, that may have been exposed to blood or body fluids etc.	20 (83)	81 (55)	95 (48)
Color code for hospital waste to be autoclaved/disinfected	13 (54)	28 (19)	45 (23)
Color code for disposal of normal waste from the hospital	13 (54)	72 (49)	86 (43)
Health hazards caused by inappropriate waste disposal	24 (100)	148 (100)	178(89)
Disposal of non-biodegradable waste (gloves, IV set, syringes, nylon sutures etc)	13 (54)	57 (39)	96 (48)
Disposal of infectious biodegradable waste (extracted teeth, human tissues, membranes, cotton dressing etc.)	13 (54)	59 (40)	67 (34)

Table 4 revealed that all components for monitoring adherence are statistically significant in influencing adherence to waste management practice. The table depicts that, all the elements assessing adherence to practice are below 50% viz; following of colour codes during disposal having 43%, steps for exposure with infected blood/body fluid has 27%, availability of waste disposal charts 36%, while having a dedicated facility manager and a committee responsible for monitoring waste generated is 22% and 28% respectively. The factors that are militating to adherence to standard hospital waste management practice in the hospital show that all the challenges are statistically significant and have the likelihood of influencing adherence to waste management practice (p<0.05). The table showed that only 31% of the respondents affirmed that there are guidelines available in their facility, with 30% responding positively that there are rules and regulations guiding the disposal of wastes in the absence of the guidelines. Forty-six percent of the respondents believed that family members can offer some form of assistance in the management of waste in the hospital towards ensuring a safe environment (Table 5). Most of the respondents, 79%, do not have color-coded containers readily available for use in their various facilities. Training on hospital waste management is very low among the respondents, as only 29% have ever attended any form of training on hospital waste management, with 15% affirming that it is not necessary for them to attend such training. 44% of the respondents make use of personal protective equipment (PPE) during waste collection and disposal, with most facilities having no dedicated waste manager, only 22% of the respondents affirmed to having a dedicated person for the management of waste. The staff to patient ratio is 23% in all the departments assessed with the questionnaire.

**Table 4:** components of adherence to hospital waste management

Variables	Good knowledge (n=371)	Poor knowledge (n=371)	Test statistic (x^2^)	p-value
Color coding waste disposal during clinical postings	158 (43)	213 (57)	8.15	0.04*
Steps to be followed after an exposure with infected blood/body fluid and contaminated sharps	101 (27)	270 (73)	76.98	0.01*
Presence of hospital waste disposal charts in your department	132 (36)	239 (64)	30.85	0.01*
Presence of facility dedicated waste manager	82 (22)	289 (78)	115.49	0.01*
Availability of responsible monitoring and evaluation committee of waste generated and disposed in your facility	105 (28)	266 (72)	69.86	0.01*

* Statistically significant difference

**Findings from Key Informant Interview (KII) perception/opinion on adherence to recommended practices:**most of the respondents mentioned lack of knowledge of standard practices, unavailability of waste management tools, and lack of sensitization and staff training as some of the major challenges affecting compliance by healthcare workers in adhering to the recommended hospital waste management practices. An informant said, *“quarterly training and sensitization of hospital staff irrespective of cadre will go a long way to improve positive staff attitude towards waste management.”*

**Factors associated with adherence:**most of the informants are of the opinion that factors such as the presence of a dedicated waste management team, the waste management plan for the hospital, and policies and regulations are directly associated with adherence to recommended hospital waste management practices by healthcare workers. An informant stated that the *“presence of waste disposal charts in various departments with guided policies and regulations will help healthcare workers adhere to recommended practices as it will serve as a reminder”*. Another informant said *“having a dedicated waste management team that would be responsible for monitoring and evaluation of hospital waste generation and disposal within the facility will do a lot in promoting and improving healthcare workers´ attitude thus improve compliance”*. Another informant stressed that *“prompt funding and availability of standard guidelines on waste management with available of working tools and equipment is a good booster to those involved directly with waste management.”*

## Discussion

The respondents in this study were assessed on knowledge, attitude, and practice of recommended hospital waste management. The result showed that 89% of the respondents knew about waste segregation, 62% knew how it should be disposed of, but only 40% and 47% of the respondents knew the correct sequence of segregation and how sharp objects should be disposed of respectively, indicating poor knowledge. This in general showed that the respondents have poor knowledge of recommended hospital waste management, with an average of 46% across all the eight (8) components measured. The overall proportion of participants with good knowledge showed 37% indicating poor knowledge of hospital waste management in the hospital. The data was further disaggregated based on cadre (Table 3), and it showed that the doctors´ category achieved 100% in the 6 components represented. Hence, high knowledge recorded in the doctors´ category may be attributed to training received by the doctors more than their counterparts (nurses and others). This result is similar to other studies that measured the knowledge regarding biomedical waste management, which showed that consultants, residents, and scientists respectively have more knowledge as compared to other categories [14,15]. This is an indication that people with higher education tend to have a greater awareness of waste management issues than their less-educated counterparts.

Segregation of medical waste at its source is the golden rule of hospital waste management. Knowledge of this strategy was displayed by only 40% of the respondents. The use of different color-coding bags for segregation is one of the most important parts of hospital waste management rule, and the result showed that only 20% of the participants have knowledge of autoclaved items and 25% of normal items, indicating poor knowledge, which is statistically significant to influence knowledge (p<0.05). The study also showed that only 36% of the department within the facility have waste disposal charts available, which guides the use of recommended practice for the disposal of waste. Twenty-two percent of the respondents know of a dedicated waste management team available and these members (where available) do not use protective clothing while handling waste, and also not practicing the correct use of colour coded bags. It also reveals that female health workers are more likely to adhere to recommended hospital waste management practice as compared to their male counterparts with an average of 28% having shown good adherences for all the components that were used to assessed adherence to practice, as against 19.1% for the male counterparts.

Furthermore, assessing the attitude of health workers towards standard hospital waste management practices showed 100% of doctors and 89% of nurses and other health care workers have a good knowledge of possible hazards that can be attributed to improper disposal of waste (Table 3). This result is concurrent with other studies in India and Nigeria (Akure) on the awareness status of hospital waste which showed that all the doctors were aware that improper management of healthcare waste causes different health hazards like infections (HIV/AIDS, Hepatitis B, and C), injuries, and environmental pollutions [4,16]. Another study also reported similar findings as the need to periodically acquaint the participants with the updated hospital waste management and handling rules was felt [17]. It is, therefore, not a surprise, due to the respondents´ poor knowledge, that their practice of waste management was poor. Another study found that many responses regarding knowledge indicate awareness about hygiene, but adherence to practice continues to be an issue, which suggests the need for a well-planned waste management training and program [18]. Consequently, lack of knowledge, poor attitude, and inefficient practice of proper waste management are some of the problems militating against proper hospital waste management. Display of apathy to the concept of waste management by health workers is a major stymie to the practice of waste disposal. Most of the respondents were not trained (29%), and (82%) are willing to undergo training programs on hospital waste management, which indicates that the determinants for adherence to the recommended practice lies with knowledge.

Some of the facilitators tested have been shown to significantly influence adherence to standard practice (Table 5). All the components tested have the likelihood of influencing adherence to the practice of waste management (p<0.05). The availability of guidelines for disposal of waste, set of rules and regulations for disposing of waste, assistance from family members of patients, training, and staff to patient ratio all have shown a statistically significant value (p=0.01), showcasing a strong likelihood of influencing adherence to practice. This is in line with a similar study that found the use of guidelines as a deterrent to waste management practice [8]. It also correlates with another finding, which suggests that the availability of a set of instructions influences adherence, and directly affects adherence to the practice of waste management [15]. A study in Ibadan (Nigeria) showed that non-compliance with standard precautions was more likely in settings with unskilled workers [19], which is consistent with the findings of this study, which showed that only 29% of the respondents had training on hospital waste management, making them inappropriately skilled in the act of handling hazardous hospital waste. Despite evidence from studies that showed the use of personal protective equipment was associated with a low level of occupational hazards; especially from sharps and infectious body fluids [20], it was noted that only one-third (44%) of the respondents were provided with personal protective devices, and had access to post-exposure prophylaxis to HIV following occupational exposure. The respondents argued that the lack of standard clear rules and regulations, standard committees for monitoring and evaluation, and the practices of waste disposal are some of the other factors that hinder the effective disposal of waste in the hospital.

## Conclusion

The study assessed the knowledge of health workers in MMSH hospital, their adherence to practice according to the recommended guidelines as well factors militating against adherence. Relevant issues were analysed and examined; research questions were formulated and tested. Reviews of the existing body of knowledge on the topic were related, and issues surrounding the impacts of these wastes on both the staff and the environment were reviewed among others. It was clear from the study, that the health workers are not ignorant of the effects of improper disposal of waste in the hospital to themselves and the environment but have poor knowledge of the recommended best practices of waste management, with a fair attitude and inadequate practices related to waste management. This study has brought out the urgent need to improve the knowledge about waste management to protect the environment from the negative impact of waste. The importance of training regarding hospital waste management cannot be over-emphasized, as a lack of proper and complete knowledge about hospital waste management impacts negatively on practices of appropriate waste disposal. The study equally recommends for formal training to all health workers in the hospital on waste management, identification and assigning of a focal person responsible for waste management in each unit/department, and the procurement and making available PPE kits and containers for collation and collection of waste.

### What is known about this topic

- *Improper management of hospital waste causes serious environmental problems;*
- *Environmental problems can arise from improper generation, handling, treatment, and disposal of waste;*
- *Gaps exists regarding the assessment of hospital waste management practices particularly in Nigeria and several other countries in sub-Saharan Africa*.

### What this study adds

- *Health care workers are aware of the effects of improper disposal of waste in the hospital to themselves and the environment;*
- *There is poor knowledge of the recommended best practices of waste management, with fair attitude and inadequate practices related to waste management in the hospital;*
- *The study advocated for the need to improve the knowledge about recommended waste management practice towards protecting the environment from the negative impact of waste*.
